# Efficacy of pembrolizumab for advanced/metastatic melanoma: a meta-analysis

**DOI:** 10.1515/med-2020-0110

**Published:** 2020-06-10

**Authors:** Qi Zhang, Geng-wei Huo, Hong-zhen Zhang, Ying Song

**Affiliations:** Department of Oncology, Hebei General Hospital, Shijiazhuang, Hebei, 050051, China; Department of Oncology, Jining No. 1 People’s Hospital, Jining, Shandong 272000, China; Department of Pharmacy, Jining No. 1 People’s Hospital, Jining, Shandong 272000, China

**Keywords:** pembrolizumab, melanoma, metastasis, response, survival

## Abstract

This study evaluates the efficacy of pembrolizumab for the treatment of advanced/metastatic melanoma. The literature search was conducted in electronic databases for studies that evaluated the efficacy and safety of pembrolizumab either alone or in combination with other treatments advanced/metastatic melanoma patients. Random-effects meta-analyses were performed to achieve pooled effect sizes of response and survival rates. The overall objective response rate (ORR) was 34.2% [95% confidence interval (CI): 30.4, 38.0]. However, ORR differed with respect to the history of prior systemic therapy. ORR was lower in studies with over 50% patients with prior therapy (25.5% [22.4, 28.5]) than in studies with under 50% patients with prior therapy (40.1% [34.1, 46.1]). ORR was higher in pembrolizumab monotherapy (32.9% [28.1, 37.7]) than in pembrolizumab–ipilimumab combination (27.6% [24.0, 31.2]). Overall ORR was inversely associated with visceral metastasis and prior systemic therapy. With pembrolizumab treatment, either alone or in combination, the progression-free survival (PFS) was 5.73 months; 12-, 24-, and 60-month PFS rate were 44%, 27%, and 25%, respectively; and 12-, 24-, and 60-month overall survival rates were 65%, 50%, and 41%, respectively. The percentage of AEs that led to treatment discontinuation was 13%. Pembrolizumab monotherapy is a valuable option for the treatment of advanced/metastatic melanoma patients.

## 1 Introduction

Melanoma is a tumor of melanocytes, which most commonly arise in the skin but may also appear in the uveal area and leptomeninges [1]. Major histopathological forms of melanoma are the superficial, nodular, lentigo maligna and acral lentiginous [2]. Melanoma constitutes 5.5% of all cancers. The incidence of cutaneous melanoma has increased from 14.1 to 22.7 cases per 1,00,000 individuals during 1992 to 2016 [3]. During the fourth decade of age, the incidence of melanoma is found to be higher in females, but by the age of 75 years, melanoma incidence is reported thrice in males than in females [4]. Melanoma-related mortality rates are relatively higher in fair-skinned population especially for those who live in lower latitudes [1].

Melanoma is an immunogenic tumor, and therefore, targeting immunological pathways for the development of efficacious treatments is essential [5]. For many decades, the most common treatment regimens for metastatic melanoma were the systemic immuno-stimulating cytokines such as interleukin-2 (IL-2) and interferon-alpha (IFN-α). However, metastatic melanoma poorly responds to cytokines, and the cure rate remains less than 10% [6]. With the use of immune checkpoint inhibitors, immunotherapy for melanoma has substantially improved. Ipilimumab, the first fully humanized immunoglobulin (Ig) G1 monoclonal antibody, was approved for metastatic melanoma. Ipilimumab blocks the cytotoxic T-lymphocyte antigen (CTLA)-4 to produce anticancer effects [7].

Later, the use of antibodies against the programmed cell death 1 protein (PD1) further improved the survival of melanoma patients [8]. Blockade of PD1–PDL1 interactions has been found to produce good antitumor response. Pembrolizumab is a high-affinity humanized immunoglobulin G4 monoclonal antibody against the immune checkpoint protein, PD1, on activated T cells. One of its ligands, the PD ligand 1 (PD-L1), which is expressed on tumor cells, macrophages, and dendritic cells, triggers tolerance to immune system and thence promotes tumor proliferation [9].

Many authors have reported the outcomes of the efficacy and safety of pembrolizumab either alone or in combination with other therapies in advanced melanoma patients, but outcomes vary considerably in these studies, which provides impetus for a systematic review seeking a refined evidence of pembrolizumab’s therapeutic potentials. The aim of this study is to conduct a meta-analysis of response and survival rates of advanced melanoma patients who were treated with pembrolizumab either alone or with other therapies to gain an up-to-date evidence of its efficacy and safety and to identify the factors affecting the efficacy.

## 2 Methods

### 2.1 Inclusion and exclusion criteria

Inclusion criteria for the current meta-analysis are as follows: a study that (a) investigated the efficacy and safety of pembrolizumab either alone or in combination with other related therapies for the management of advanced/metastatic melanoma patients and (b) reported the efficacy indices including objective response rate (ORR), progression-free survival (PFS), and overall survival (OS). Exclusion criteria are as follows: a study that reported (a) the outcomes of more than one anti-PD1 drugs without distinction, (b) pharmacokinetic or pharmacodynamic investigation, (c) *in vitro*, molecular, or experimental investigations, or (d) qualitative information.

### 2.2 Literature search

Google Scholar, PubMed, and Science Direct electronic databases were searched by using specific keywords and medical subject headings. Primarily, pembrolizumab–melanoma efficacy combination was used, which was then used with several other words including programmed cell death, PD1, ligand, PD-L1, response, survival, tumor, node, metastasis, TNM, B-Raf proto-oncogene (BRAF), safety, tolerability, adverse events, toxicity, and trial. Search encompassed research articles published before September 2019 in English. In addition, the bibliographies of important related papers were also screened.

### 2.3 Data and analyses

Baseline demographic, clinical, oncological, and genetical data; and study design, methodological, analytical, and outcome data of the included studies were obtained from respective research articles and were organized in datasheets. Quality assessment of the included studies was performed with New Castle–Ottawa Scale for the Quality Assessment of Cohort studies.

Response and survival rates reported by the individual studies were pooled under the random-effects model to achieve an overall effect size of each endpoint as an inverse variance weighted average of the individual study effect sizes. Statistical heterogeneity was estimated with *I* ^2^ index. Subgroup analyses were performed with regards to the combinational use of pembrolizumab and the percentage of patients with prior systemic therapy.

In meta-regression analyses, the ORR was used as a dependent variable to seek its relationships with several independent variables including follow-up duration, age, gender, tumor/node/metastasis (TNM) status, Eastern Cooperative Oncology Group performance status (ECOG PS), PD1 ligand status, BRAF status, percentage of patients with high lactic dehydrogenase (LDH) levels, and prior systemic therapy. The restricted maximum likelihood method was used for meta-regression analyses. All statistical analyses were performed using Stata software (Stata Corporation, Texas, USA).

## 3 Results

**Figure 1:**
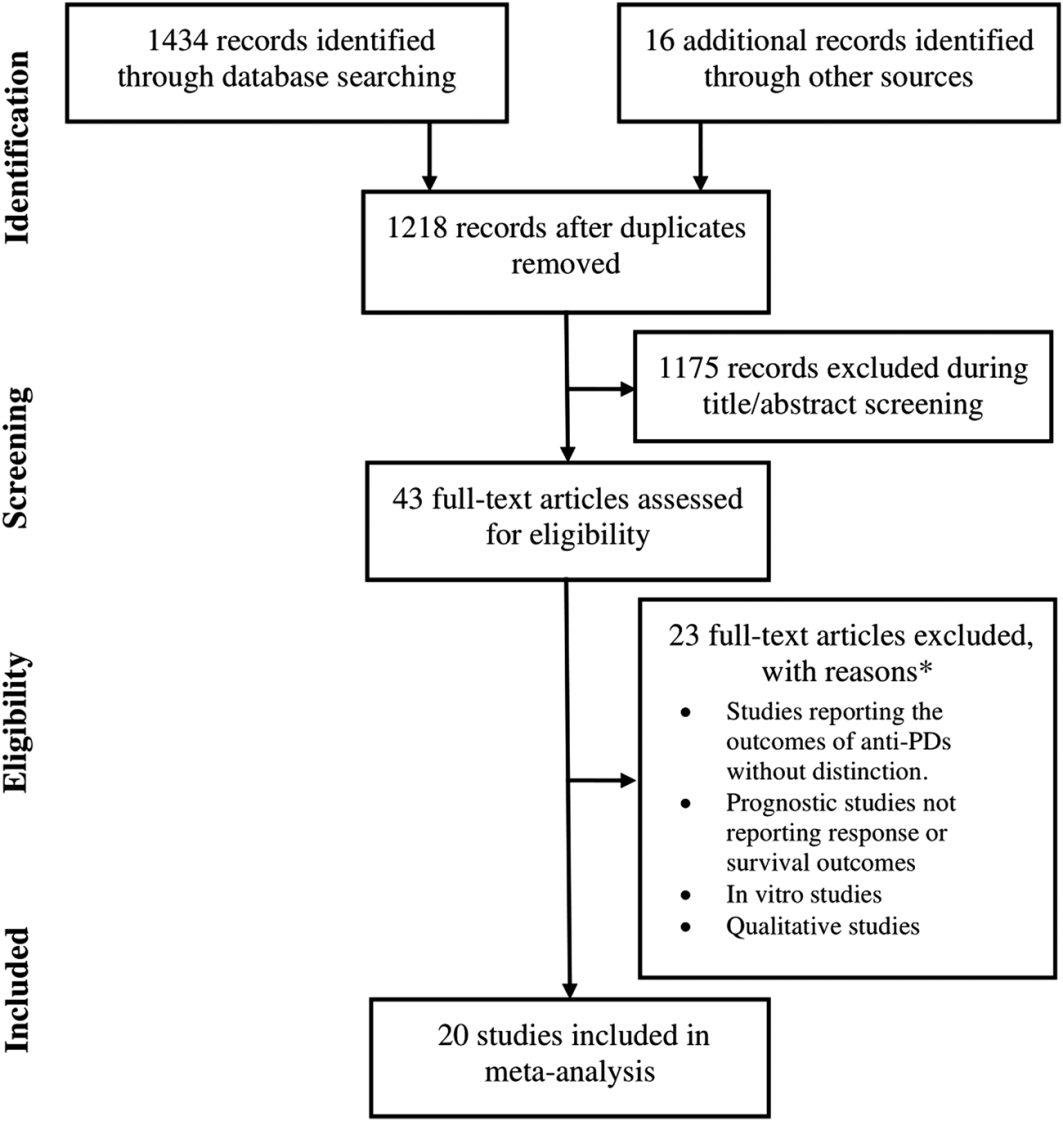
A flowchart of study screening and selection process.

Twenty studies fulfilled the eligibility criteria and thence were included in the meta-analysis (Figure 1). These studies were published in 25 research articles [10,11,12,13,14,15,16,17,18,19,20,21,22,23,24,25,26,27,28,29,30,31,32,33,34]. In these studies, 2,909 patients with advanced/metastatic melanoma were treated with pembrolizumab either alone (*n* = 2,139) or in combination with other therapies (*n* = 770). Characteristics of the included studies are presented in Tables S1a and b. The average age of these patients was 62.5 years [95% confidence interval (CI): 60.3, 64.8]. The percentage of females in this population was 39% [95% CI: 36, 40]. The quality of the included studies was moderate to high in general (Table S2).

The average median follow-up duration in these studies was 25.6 months [95% CI: 20.4, 30.8]. Of all patients, 41.3% [95% CI: 40.6, 40.0] had prior systemic treatment for melanoma. The percentage of patients with BRAF mutation was 33% [95% CI: 25, 41], whereas 42.7% [95% CI: 34.8, 50.5] patients were PD-L1 positive. Percentages of patients with M0, M1a, M1b, and M1c TNM stages were 5.0% [95% CI: 4.2, 5.8], 10.5% [95% CI: 9.0, 12.0], 17.9% [95% CI: 15.4, 20.4], and 69.4% [95% CI: 66.3, 72.6], respectively. Among these patients, 21.8% [95% CI: 19.9, 23.8] had metastases in the brain. Percentages of patients with ECOG PS 0 and ECOG PS 1 were 65.6% [95% CI: 60.1, 71.1] and 28.7% [95% CI: 26.1, 31.3], respectively.

### 3.1 Response rate

**Figure 2:**
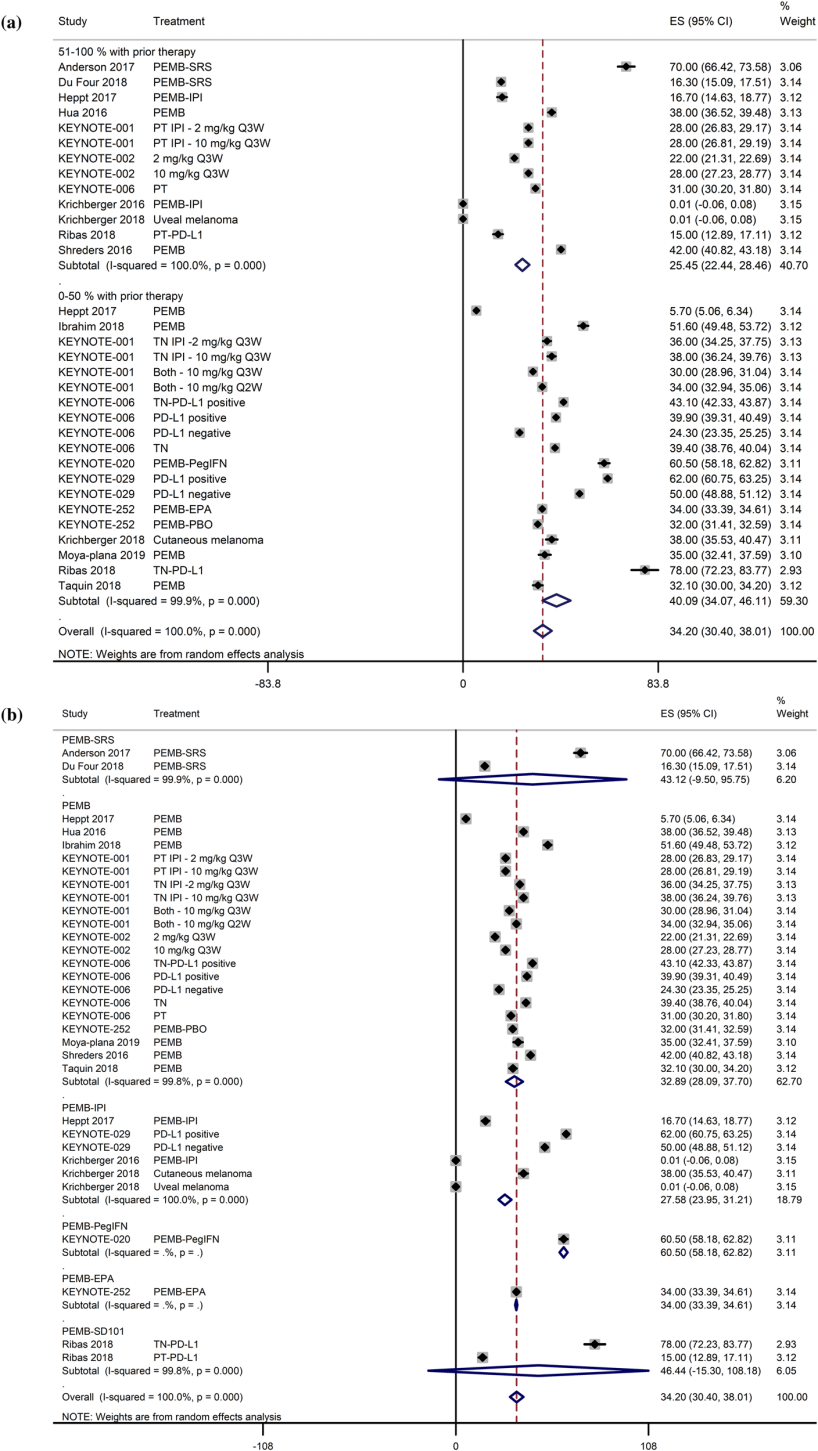
(a) A forest graph showing the pooled effect sizes of ORR (ES; effect size with 95% CI) with regards to the percentage of patients with prior therapy. (b) A forest graph showing the pooled effect sizes of ORR (ES; effect size with 95% CI) with regards to the combination of pembrolizumab treatment.

Response was achieved in 12.1 weeks [95% CI: 12.0, 12.2], and the response duration was not reached within the follow-up durations of most studies. The overall ORR was 34.2% [95% CI: 30.4, 38.0]. However, ORR differed with respect to the history of prior systemic therapy. The ORR was substantially lower in studies with over 50% patients with prior systemic therapy (25.5% [95% CI: 22.4, 28.5]) than in studies with less than 50% patients with prior systemic therapy (40.1% [95% CI: 34.1, 46.1; Figure 2a). The ORR was higher for pembrolizumab monotherapy (32.9% [95% CI: 28.1, 37.7]) than for pembrolizumab–ipilimumab combination (27.6% [95% CI: 24.0, 31.2]; Figure 2b).

The complete remission (CR) and partial remission (PR) rates were also lower in studies with over 50% patients with prior systemic therapy than in studies with less than 50% patients with prior systemic therapy (Figures S1a and b). The CR rate was slightly higher for pembrolizumab monotherapy than pembrolizumab–ipilimumab combination (Figure S2a), whereas the PR rate was similar for pembrolizumab monotherapy and pembrolizumab–ipilimumab combination (Figure S2b). The stable disease (SD) and progressive disease (PD) rates were higher in studies with over 50% patients with prior systemic therapy than in studies with less than 50% patients with prior systemic therapy (Figures S3a and b). The SD rate was lower with pembrolizumab monotherapy than with pembrolizumab–ipilimumab combination (Figure S4a), whereas the PD rate was slightly higher with pembrolizumab monotherapy than with pembrolizumab–ipilimumab combination (Figure S4b).

### 3.2 Factors affecting the objective response rate

In the meta-regression analyses, independently, the overall ORR was significantly positively associated with the percentage of patients with TNM M1a stage, TNM M1b stage, and ECOG PS 0 but was significantly inversely associated with the percentage of patients with TNM M1c stage, high LDH levels, and prior systemic therapy (Table 1). In multivariate metaregression analyses with TNM M1c, high LDH levels, and prior therapy as covariates, only TNM M1c was significantly inversely associated with the overall ORR. Moreover, in multivariate analyses with TNM M1a, TNM M1b, and ECOG PS 0 as covariates, only TNM M1b was significantly positively associated with the overall ORR.

**Table 1:** Independent relationships of ORR with explanatory variables

Explanatory variable	Metaregression coefficient [95% CI]	*P*	Datasets
Age (years)	0.87 [−0.641, 2.390]	= 0.246	29
Females (%)	−0.241 [−1.078, 0.597]	= 0.561	32
Follow-up duration (months)	−0.037 [−0.735, 0.661]	= 0.914	29
% patients with TNM M0	0.018 [−0.276, 0.314]	= 0.892	14
% patients with TNM M1a	1.201 [0.464, 1.938]	= 0.004	14
% patients with TNM M1b	1.259 [0.414, 2.104]	= 0.007	14
% patients with TNM M1c	−0.857 [−1.251, −0.463]	<0.00001	19
% patients with brain metastasis	0.043 [−0.219, 0.306]	= 0.736	23
% patients with 0 ECOG PS	0.596 [0.133, 1.060]	= 0.014	23
% patients with 1 ECOG PS	−0.180 [−0.969, 0.610]	= 0.644	28
% patients with BRAF mutations	−0.110 [−0.565, 0.345]	= 0.622	26
% patients with high LDH levels	−0.355 [−0.777, 0.066]	= 0.094	26
% patients with PD-L1 positivity	0.119 [−0.137, 0.375]	= 0.326	12
% patients with prior therapy	−0.240 [−0.392, −0.088]	= 0.003	32

### 3.3 Survival

**Figure 3:**
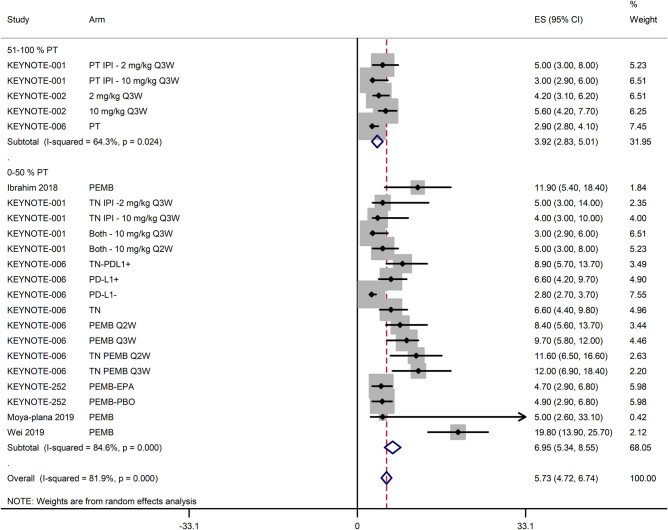
A forest graph showing the pooled PFS (ES; effect size with 95% CI) with regards to the percentage of patients with prior therapy.

**Figure 4:**
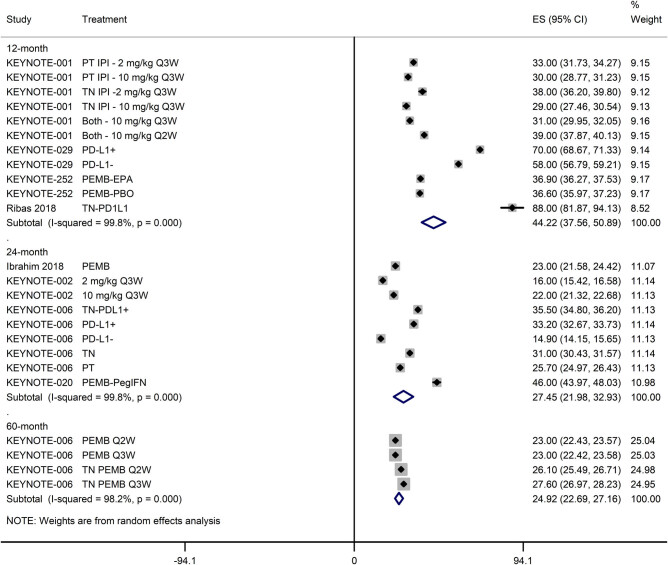
A forest graph showing the pooled 12-, 24-, and 60-month PFS (ES; effect size with 95% CI).

The PFS of melanoma patients treated with pembrolizumab either alone or in combination with other therapies was 5.73 months [95% CI: 4.72, 6.74]. However, it was lower in studies with over 50% patients with prior therapy (3.92 months [95% CI: 2.83, 5.01]) than in studies with under 50% patients with prior therapy (6.95 months [95% CI: 5.34, 8.55]; Figure 3). The 12-, 24-, and 60-month PFS rates of patients treated with pembrolizumab either alone or in combination with other therapies were 44.22% [95% CI: 37.56, 50.89], 27.45% [95% CI: 21.98, 32.93], and 24.92% [95% CI: 22.69, 27.16], respectively (Figure 4).

**Figure 5:**
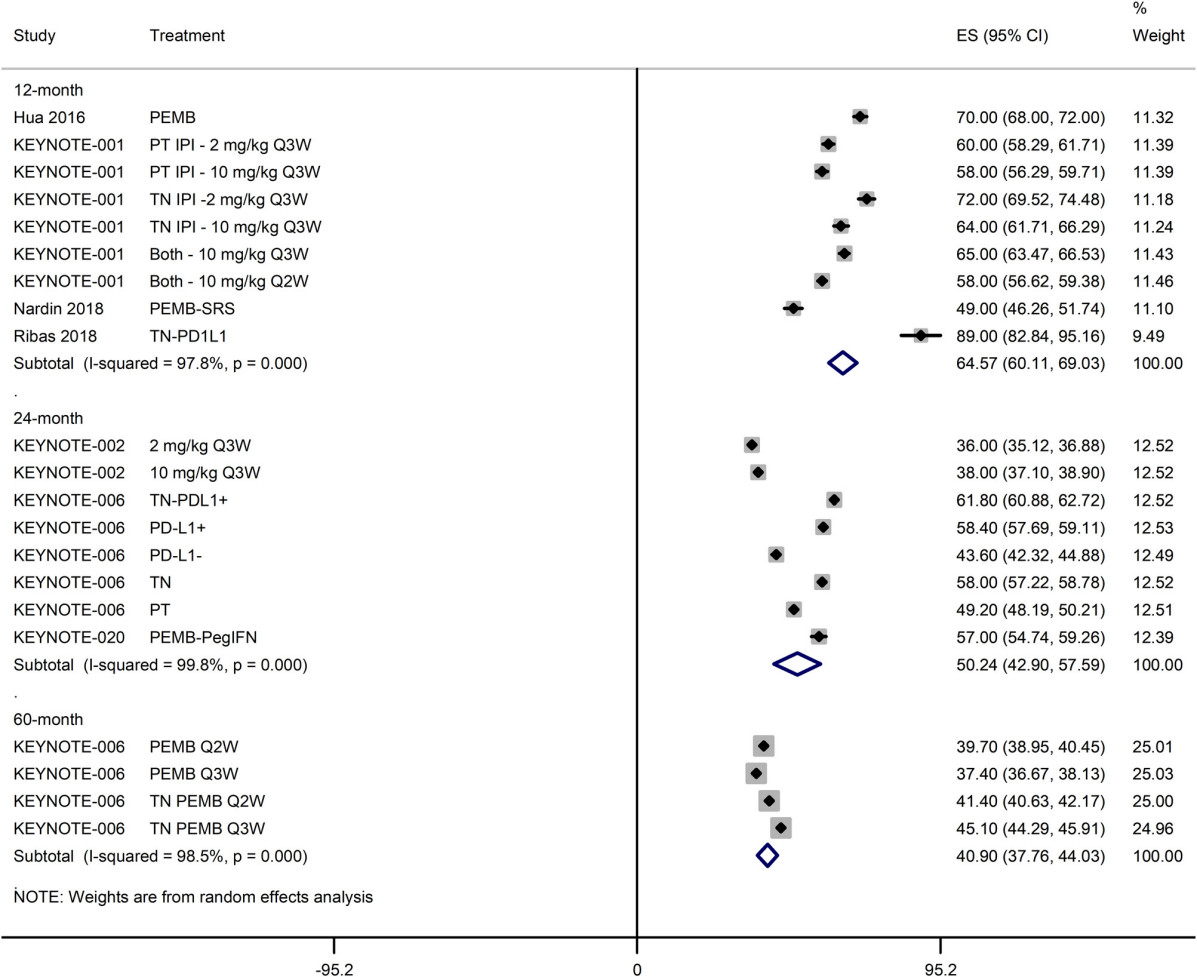
A forest graph showing the pooled 12-, 24-, and 60-month OS (ES; effect size with 95% CI).

The OS was not achieved within the follow-up durations of many studies. For the remaining of the studies (*n* = 6), the OS was 20.16 months [95% CI: 16.04, 24.27], which was lower in studies with over 50% patients with prior systemic therapy (15.15 months [95% CI: 11.97, 18.34]) than in studies with under 50% patients with prior systemic therapy (25.58 months [95% CI: 19.23, 31.92]; Figure S5). The 12-, 24-, and 60-month OS rates of patients treated with pembrolizumab either alone or in combination with other therapies were 64.57% [95% CI: 60.11, 69.03], 50.24% [95% CI: 42.90, 57.59], and 40.90% [95% CI: 37.76, 44.03], respectively (Figure 5).

### 3.4 Safety analysis

Major adverse events observed by the included studies are summarized in Table 2. The percentage of AEs that led to discontinuation of treatment was 13.0% [95% CI: 10.5, 15.6]. Fatigue, headache, pruritis, rash, nausea/vomiting, diarrhea, vitiligo, and arthralgia were the most frequent AEs that were observed in two or more studies. AEs reported by less than two studies included abdominal pain, alopecia, asthenia, constipation, dyspnea, eczema, high amylase, high lipase, hypersensitivity, hypoalbunemia, hypocalcemia, hyponatremia, hypophosphatemia, leukopenia, malaise, perilesional edema, seizures, and thrombosis.

**Table 2:** Adverse events observed during pembrolizumab treatment reported by the included studies

Adverse event	Incidence (weighted, averaged percentage)	Number of studies
AEs leading to discontinuation	13.02 [10.49, 15.55]	7
Anemia	4.79 [3.46, 6.12]	4
Anorexia	14.48 [9.43, 19.53]	3
Arthralgia	11.28 [9.32, 13.25]	7
Colitis	5.16 [3.62, 6.70]	4
Diarrhea	15.69 [12.58, 18.80]	5
Fatigue	29.92 [23.43, 36.41]	5
Headache	29.18 [21.31, 37.04]	3
High ALT	7.94 [5.42, 10.46]	2
High AST	7.74 [5.82, 9.66]	2
Hyperthyroidism	5.66 [1.95, 9.37]	2
Hypophosphatemia	5.70 [3.45, 7.96]	2
Hypothyroidism	8.42 [5.60, 11.23]	7
Lymphopenia	7.55 [5.44, 9.67]	2
Myalgia	7.43 [5.74, 9.12]	4
Nausea/vomiting	16.36 [12.85, 19.87]	7
Neutropenia	3.37 [2.49, 4.25]	3
Pneumonitis	2.00 [1.18, 2.82]	2
Pruritis	22.91 [19.23, 26.58]	7
Rash	18.17 [15.49, 20.85]	7
Renal	0.47 [0.21, 0.72]	3
Thrombocytopenia	0.42 [0.31, 0.53]	2
Vitiligo	11.82 [10.68, 12.97]	4

Abbreviations: AEs, adverse events; ALT, alanine transaminase; AST, aspartate transaminase.

## 4 Discussion

In this meta-analysis, we have found that the pembrolizumab treatment either alone or in combination with other therapies led to the ORR, PFS, and OS of approximately 34%, 5.7 months, and 20.3 months, respectively, which were lower in previously treated patients than in naïve patients. The overall ORR was higher for pembrolizumab monotherapy than pembrolizumab–ipilimumab combination. Independently, the overall ORR was significantly inversely associated with TNM M1c and the percentage of patients with prior therapy but was positively associated with ECOG PS 0 score. Two-year OS rate of pembrolizumab either alone or in combination with other therapies was approximately 50%.

The immune-checkpoint blockade is a type of passive immunotherapy to enhance innate antitumor response by blocking interactions between T-lymphocytes and neoplasm. Pembrolizumab blocks the interaction between PD1 and PD-L1 to make melanoma cells vulnerable to the T-lymphocyte attack. Because PD-L1 is highly expressed in at least 50% of melanomas, targeting PD1–PDL1 pathway is now foreseen as a promising therapeutic target [35].

A combined therapy with ipilimumab and nivolumab resulted in better response and survival outcomes than their monotherapies; however, this was associated with higher toxicity [36,37]. In the present study, we have found that the response rate of pembrolizumab monotherapy was higher than the response rate of pembrolizumab–ipilimumab combination, which shows that the superiority of pembrolizumab monotherapy over its combinational use with ipilimumab is promising for melanoma patients. KEYNOTE-006 authors have supported the use of pembrolizumab monotherapy as the standard of care for advanced melanoma based on their study findings [19].

Mechanistically, anti-PD1 and anti-CTLA4 monotherapies manifest many distinct effects, which differ also from their combinational use. *In vivo* studies have shown that CTLA4 blockade leads to T cell proliferation and PD1 blockade induces several genes involved in cytolysis and natural killer cell function [38]. The anti-PD1 activity makes T cells and myeloid-derived suppressor cells more available in tumors. Such a pronounced effect is observed in CD8(+) effector memory T-cell expansion in biopsies of patients who responded to therapy [39].

In a study in which patients were treated with pembrolizumab when they progressed on ipilimumab, ipilimumab PFS was related to pembrolizumab outcomes, so that the patients with prolonged PFS on ipilimumab also had higher response, PFS, and OS rates with pembrolizumab and patients who progressed earlier on ipilimumab also exhibited a worse response to pembrolizumab. The authors suggested that this may indicate the presence of “immune-responsive” and “immune-resistant” phenotypes in melanoma patients, which may require targeting each category separately with appropriate therapies [32]. It has been suggested that trials with longer follow-up are important to determine whether there exists a “plateau effect” in overall survival after pembrolizumab treatment [22]. In the present study, we have noticed that although 24-month and 60-month survival differed more from 12-month survival, the difference was less between 24-month and 60-month survival, which may support the notion of the existence of a “plateau effect” in the survival of melanoma patients after pembrolizumab therapy.

We have found that although TNM M1a and M1b stages were positively associated with the ORR, TNM M1c was inversely associated with the ORR. Because these stages represent metastases in various anatomical sites, i.e., distant skin, subcutaneous, or nodal metastases (M1a), pulmonary metastases (M1b), and visceral metastases (M1c), our results suggest that visceral metastases lead to poor prognosis. However, in the population of the present study, percentages of patients with M0, M1a, M1b, and M1c were 5.0%, 10.5%, 17.9%, and 69.4%, respectively. This imbalanced distribution might have affected the overall analysis.

Some limitations of the present study are important to mention. High statistical heterogeneity in most analyses is an important factor. Variations in designs, combinational use of pembrolizumab, tumor stage, PDL1 +/− status, and prior treatment history across the included studies could have played roles in contributing heterogeneity. Thus, such factors might have caused high *I* ^2^ values. Another factor was that combinational use could be studied with considerable power only in pembrolizumab–ipilimumab. Thus, the outcomes presented herein are majorly derived from pembrolizumab monotherapy.

In conclusion, a population of advanced/metastatic melanoma patients, of whom 33% had BRAF mutation, 43% were PD-L1 positive, and 41% had prior systemic therapy, were followed up for approximately for 26 months, and pembrolizumab treatment either alone or in combination with other agents led to the ORR, PFS, and OS of approximately 34%, 5.7 months, and 20.3 months, respectively, all of which were higher in treatment in naïve patients. The response rates were higher for pembrolizumab monotherapy than pembrolizumab–ipilimumab combination. Two-year OS rate was approximately 50% in this population. These results suggest that the superiority of pembrolizumab monotherapy over its combinational use with ipilimumab is promising for melanoma patients.
